# Machine learning for fast identification of bacteraemia in SIRS patients treated on standard care wards: a cohort study

**DOI:** 10.1038/s41598-018-30236-9

**Published:** 2018-08-15

**Authors:** Franz Ratzinger, Helmuth Haslacher, Thomas Perkmann, Matilde Pinzan, Philip Anner, Athanasios Makristathis, Heinz Burgmann, Georg Heinze, Georg Dorffner

**Affiliations:** 1 Department of Laboratory Medicine, Division of Medical and Chemical Laboratory Diagnostics, Medical University of Vienna, Vienna, Austria; 2 Center for Medical Statistics, Informatics and Intelligent Systems, Section for Artificial Intelligence and Decision Support, Medical University of Vienna, Vienna, Austria; 3 Department of Laboratory Medicine, Division of Clinical Microbiology, Medical University of Vienna, Vienna, Austria; 4 Department of Medicine I, Division of Infectious Diseases and Tropical Medicine, Medical University of Vienna, Vienna, Austria; 5 Center for Medical Statistics, Informatics, and Intelligent Systems, Section for Clinical Biometrics, Medical University of Vienna, Vienna, Austria

## Abstract

Bacteraemia is a life-threating condition requiring immediate diagnostic and therapeutic actions. Blood culture (BC) analyses often result in a low true positive result rate, indicating its improper usage. A predictive model might assist clinicians in deciding for whom to conduct or to avoid BC analysis in patients having a relevant bacteraemia risk. Predictive models were established by using linear and non-linear machine learning methods. To obtain proper data, a unique data set was collected prior to model estimation in a prospective cohort study, screening 3,370 standard care patients with suspected bacteraemia. Data from 466 patients fulfilling two or more systemic inflammatory response syndrome criteria (bacteraemia rate: 28.8%) were finally used. A 29 parameter panel of clinical data, cytokine expression levels and standard laboratory markers was used for model training. Model tuning was performed in a ten-fold cross validation and tuned models were validated in a test set (80:20 random split). The random forest strategy presented the best result in the test set validation (ROC-AUC: 0.729, 95%CI: 0.679–0.779). However, procalcitonin (PCT), as the best individual variable, yielded a similar ROC-AUC (0.729, 95%CI: 0.679–0.779). Thus, machine learning methods failed to improve the moderate diagnostic accuracy of PCT.

## Introduction

Bacteraemia is a frequent and challenging condition with a mortality rate ranging between 13% and 21%^1–3^. Risk factors for bacteraemia are advanced patient age, urinary or indwelling vascular catheter, chemotherapy or immunosuppressive therapies and co-morbidities such as malignancies^4–7^. A timely diagnosis is pivotal for the survival of bacteraemic patients, as these patients require prompt treatment with the appropriate antibiotics^8,9^.

Although blood culture (BC) analysis is regarded as the gold standard in bacteraemia diagnostics, the clinical decision as to who should receive BC analysis is not trivial. Furthermore, BC analysis needs a median of three days for a positive report and singularly taken BC often lacks diagnostic sensitivity^10,11^. Despite profound knowledge about its pre-test probability, which is severely affected by the infection site, the true positive result rate of BC analysis for recognized pathogens ranges between 4% and 7%^12–14^. Moreover, the proportion of false positive BC results related to contaminations is in a comparable range of up to over 8% of all BC analyses^14–16^. Generally, these flaws in the utilization of BC analysis have a fundamental economic impact, with estimated costs ranging between $6,878 and $7,502 for a single false positive BC result^17–19^.

Consequently, physicians are frequently faced with diagnostic uncertainties^20^. Biomarkers or prediction tools with a high negative predictive value (NPV), enabling the exclusion of bacteraemia, are highly desirable to increase the cost-effectiveness of microbiological tests. Procalcitonin (PCT) is considered as the best biomarker for detecting bacteraemia, with a pooled sensitivity of 76% (95% confidence interval (CI): 72–80%) and a pooled specificity of 69% (95% CI: 64–72)^21^.

In the current study, machine learning algorithms were applied to data obtained by a prospective cohort study with the goal to improve the diagnostic performance of PCT for identifying patients fulfilling two or more systemic inflammatory response syndrome (SIRS) criteria but without the need for BC analysis.

## Results

### Study population and available data

Data of 466 SIRS patients was available for predictive model estimation. Among them, 134 patients (28.8%) suffered from microbiologically confirmed bacteraemia, 195 patients (41.8%) presented with an infection but without bacteraemia and 137 patients (29.4%) presented with a SIRS syndrome which was not related to any infection. The in-hospital mortality was 11.1% (*n* = 52) in our cohort.

In total, 71 patients fulfilled four SIRS criteria, 213 patients presented three SIRS criteria and 182 patients presented with two SIRS criteria. Among the study population, a considerable proportion suffered from oncological or hemato-oncological diseases (40.6%, *n* = 189). A total of 86 patients received antibiotic therapy (18.5%) before blood sample taking. Clinical and laboratory data of the study population are presented in Table 1 and Table 2. Most common infection foci were respiratory tract infections (*n* = 94, 14.9% bacteraemia rate), urinary tract infections (*n* = 51, 23.5% bacteraemia rate) and gastrointestinal system infection (*n* = 50, 40.0% bacteraemia rate, see: Supplementary Table 1). In 34 bacteraemic patients, no primary infection focus was found. The distribution of pathogens detected in BC and in the SeptiFast MGRADE test (Roche Diagnostics GmbH, Mannheim, Germany) is presented in Supplementary Table 2. More than one pathogen was detected in 13 patients.

**Table 1:** Clinical data of study participants.

Feature	Missing	No bacteraemia (*n* = 332)	Bacteraemia (*n* = 134)	p–value
Age	0.0%	56.7 (41.9–69.0)	60.1 (45.3–69.2)	0.206
BMI	0.0%	24.8 (21.6–28.8)	24.8 (20.8–27.6)	0.183
Sex	0.0%	192: 140 (57.8%: 42.2%)	71: 63 (53.0%: 47.0%)	0.354
AB naïvety	0.0%	264 (79.5%)	116 (86.6%)	0.087
Catheter	0.0%	85 (25.6%)	45 (33.6%)	0.088
Post–surgical	0.0%	22 (6.6%)	7 (5.2%)	0.777
Neoplasm	0.0%	129 (38.9%)	60 (44.8%)	0.252
HBR	0.0%	99 (92–107)	100 (91–110)	0.195
RR	0.0%	20 (16–24)	21 (16.5–24)	0.094
BT	0.0%	38.4 (38–38.9)	38.5 (38.1–39.1)	0.005*
SIRS–No#	0.0%	130:155:47	52:58:24	0.558
COPD	0.0%	42 (12.8%)	12 (9.0%)	0.268
Asplenia	0.0%	8 (2.4%)	2 (1.5%)	0.731
Dysuria	0.0%	28 (8.5%)	15 (11.2%)	0.379
Dialysis	0.0%	15 (4.5%)	7 (5.2%)	0.810
Diabetes	0.0%	57 (17.2%)	25 (18.7%)	0.689

*significant after applying the Bonferroni-Holm correction, BMI = body mass index, AB naivety = antibiotics prior to blood culture sampling: yes:no, HBR = heart beat rate, RR = respiration rate, BT = body temperature, COPD = Chronic obstructive pulmonary disease.

**Table 2:** Laboratory data analysed in the study.

Feature	Unit	Missing	No bacteraemia	Bacteraemia	p–value	ROCs (95%CI)
PCT	ng/ml	1.5%	0.3 (0.1–1.0)	1.6 (0.4–5.4)	<0.001*	0.729 (0.679–0.779)
CRP	mg/dl	0.4%	12.9 (7.9–20.4)	15.0 (9.6–22.8)	0.020	0.569 (0.512–0.626)
LBP	µg/ml	1.1%	23.1 (15.6–35.4)	29.7 (19.7–44.25)	<0.001*	0.610 (0.553–0.667)
IL-6	pg/ml	1.7%	42.8 (19.2–99.9)	49.6 (28.9–130.0)	0.028	0.566 (0.508–0.623)
Fib	mg/dl	6.0%	607 (446–752)	613 (490–714)	0.875	0.505 (0.447–0.563)
SI	µg/dl	1.7%	26.0 (14.8–57.0)	21.0 (12.3–46.8)	0.042	0.561 (0.502–0.619)
TP	g/l	2.4%	61.6 (55.8–67.5)	60.5 (53.2–65.8)	0.062	0.556 (0.498–0.614)
ALAT	U/L	1.9%	25.0 (15.0–45.0)	33.0 (18.0–64.0)	0.005	0.585 (0.526–0.643)
Alb	g/l	2.4%	31.3 (27.9–35.1)	29.5 (25.6–33.1)	<0.001*	0.603 (0.546–0.673)
Bili	mg/dl	3.9%	0.6 (0.5–1.0)	0.8 (0.6–1.4)	<0.001*	0.616 (0.558–0.673)
γ–GT	U/L	2.4%	63 (30–130)	102 (44–260)	<0.001*	0.617(0.559–0.675)
Crea	mg/dl	0.4%	0.9 (0.8–1.3)	0.9 (0.8–1.3)	0.707	0.511 (0.452–0.570)
LDH	U/L	4.3%	220 (168–312)	200 (157–286)	0.161	0.543 (0.485–0.602)
Hb	g/dl	2.4%	10.0 (9.0–11.7)	10.0 (9.1–10.9)	0.340	0.529 (0.473–0.584)
Plt	G/l	2.4%	208 (116–326)	190 (134–279)	0.426	0.524 (0.457–0.580)
WBC	G/l	2.4%	8.7 (5.4–13.6)	8.7 (5.5–12.6)	0.629	0.486 (0.428–0.543)
NeuR	%	7.1%	75.5 (63.9–82.8)	79.5 (67.2–86.6)	0.021	0.570 (0.510–0.631)
EosR	%	4.3%	0.9 (0.2–2.5)	0.8 (0.2–1.9)	0.308	0.531 (0.472–0.589)
IL-10**	pg/ml	0.0%	2.2 (1.4–4.6)	3.2 (1.7–7.3)	0.002	0.589 (0.532–0.645)
IL-17a**	pg/ml	0.0%	0.8 (0.0–3.1)	2.7 (0–7.5)	<0.001*	0.601 (0.542–0.660)
MIP-1b**	pg/ml	0.0%	52.1 (29.7–82.5)	72.05 (43.6–134.7)	<0.001*	0.615 (0.557–0.673)

*significant after applying the Bonferroni-Holm correction; CRP = C-reactive protein, LBP = lipopolysaccharide binding protein, PCT = procalcitonin; IL-6 = interleukin-6, Fib = fibrinogen according to Clauss, SI = serum iron, TP = total protein, ALAT = alanine transaminase, Alb = albumin, Bili = bilirubin, γ-GT = gamma-glutamyl transpeptidase, Crea = creatinine, LDH = lactate dehydrogenase, Hb = haemoglobin, Plt = platelets, WBC = white blood cell counts, NeuR = relative proportion of neutrophils, EosR = relative proportion of eosinophils, IL-10 = interleukin-10, IL-17a = interleukin-17a, MIP-1b = macrophage inflammatory protein-1β, **not in routine use.

**Figure 1:**
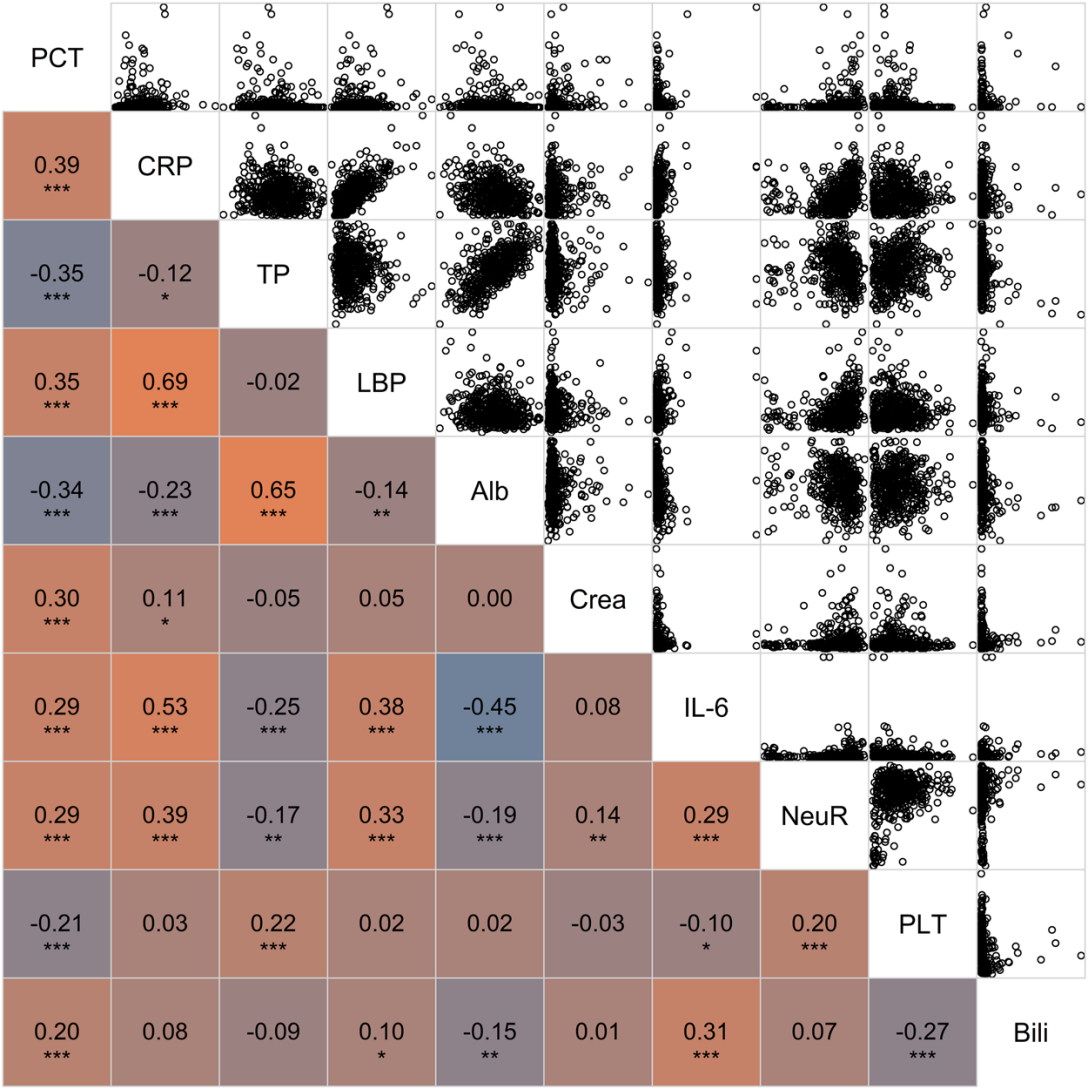
Correlogram of features with the highest correlation to PCT. The labelling of the x and y axis is presented in the diagonal. Following parameters are displayed: PCT = procalcitonin, CRP = C-reactive protein, TP = total protein, LBP = lipopolysaccharide binding protein, Alb = albumin, Crea = creatinine, IL-6 = interleukin-6, NeuR = relative proportion of neutrophils, Plt = platelets, Bili = bilirubin; Spearman correlation coefficient is presented in the left lower part of the correlogram p-values are denoted as following: ***<0.0001, **<0.001,*<0.05, in the right upper part of the correlogram scatterplots of the presented features are shown.

The best individual variable for predicting bacteraemia was procalcitonin (PCT) with a median area under the receiver operating curve (ROC-AUC) of 0.729 (95%CI: 0.679–0.779). The highest absolute correlation coefficients between PCT and other variables used for model training were found for C-reactive protein (CRP), total protein (TP) and lipopolysaccharide-binding protein (LBP; r_s_ = 0.39, −0.35 and 0.35 respectively, see Fig. 1). As non-routinely used inflammation markers, several cytokines including IL-10, IL-17a and MIP-1b were analysed, which presented a low to moderate predictive capacity with a ROC-AUC ranging between 0.589 and 0.615. Interestingly, CRP, as a widely used infection marker, presented with a low predictive capacity (ROC-AUC: 0.569, 95%CI: 0.512–0.626), while several liver-related blood variables were significantly elevated in bacteraemic SIRS patients (e.g. bilirubin, gamma-glutamyl transpeptidase (γ-GT) or alanine transaminase (ALAT), see Table 2).

**Figure 2:**
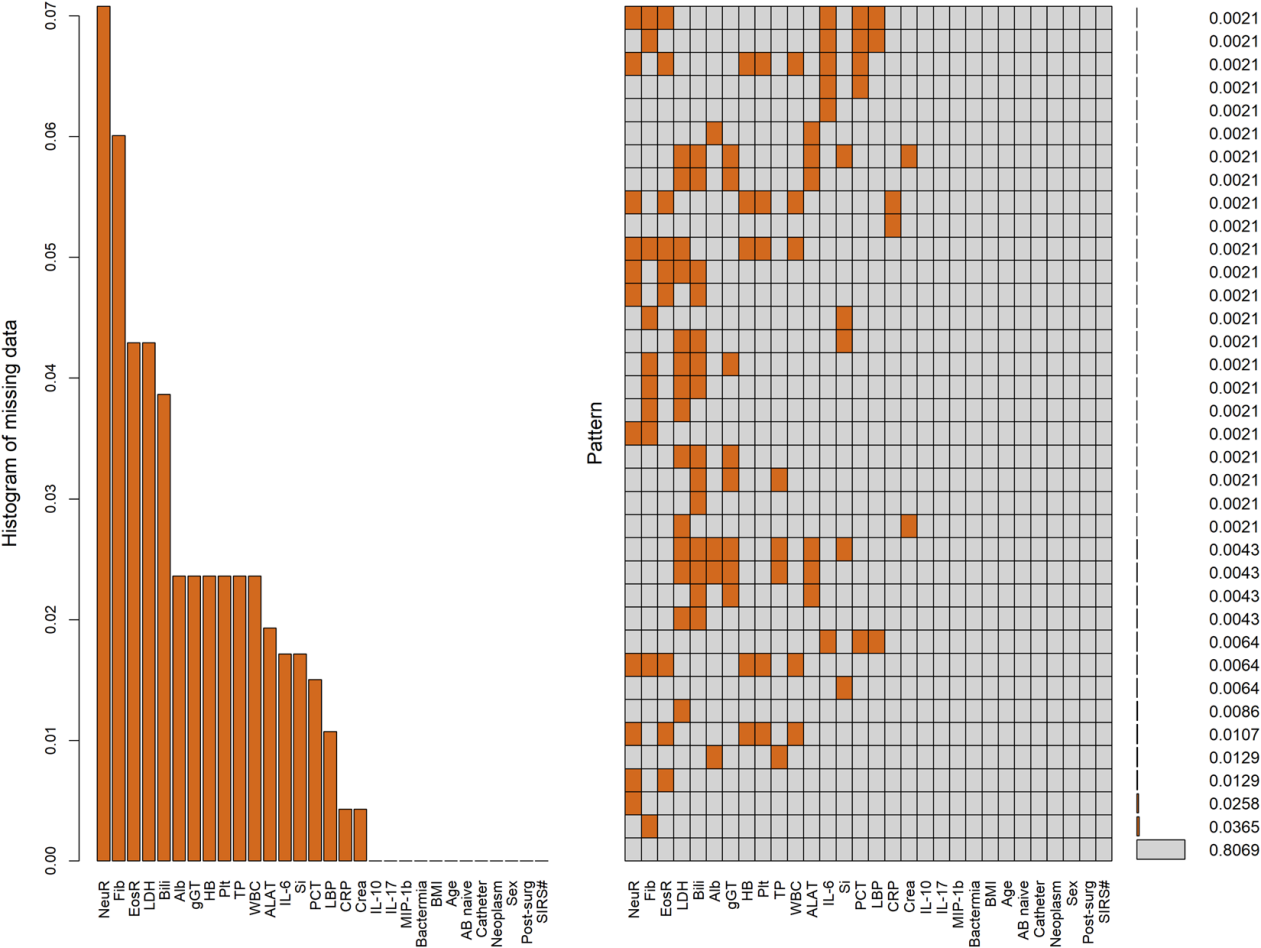
Missing data aggregation plot. left = distribution of missing data, shown in percentage, right = missing pattern analysis (aggregation missingness plot, VIM package), percentages of missing patterns are displayed on the right side, 81% of the total study population had no missing values.

In a next step, patterns of missing variables were analysed (see Fig. 2). The relative proportion of neutrophils (NeuR) and eosinophils (EosR) as well as fibrinogen (Fib) showed the highest amount of missing data (7%, 4% and 6% missingness respectively). When assessing distinct missingness patterns, Fib alone (3.7% of all patients) and NeuR alone (2.5% of all patients) were the most prominent patterns. Missing data was imputed using MI, generating 50 complete data sets. The imputed data sets differed in their imputed values, resembling the uncertainty of the missing values. After MI, imputed datasets were split into a training set and a test set using a 80:20 ratio and the splitting step was repeated ten times with each complete data set.

### Model training and test set validation

As described in the Methods section, models were tuned using a 10-fold CV schema (repeated ten times). In test set validation (repeated ten times), the best ROC-AUC was found using the random forest (*rf)* approach with a 0.738 ROC-AUC (95%CI: 0.606–0.870), while the neural network model (*nn)* resulted in 0.698 ROC-AUC (95%CI: 0.549–0.857) and the elastic net regression (en) approach yielded 0.654 ROC-AUC (0.493–0.815). All models lead to a similar or lower performance than PCT, as the best individual variable, with 0.729 ROC-AUC (95%CI: 0.679–0.779).

When restricting the model training and validation process to those SIRS patients without any antibiotic therapy before blood culture taking, all three ML approaches presented a similar predictive capacity. Table 3 presents data in comparison to PCT as a reference. Moreover, models were also established for patients with two, three or four SIRS criteria fulfilled (see Table 3). Best results were found in patients with three SIRS criteria fulfilled, in that the *rf* approach resulted in 0.781 ROC-AUC (95% CI: 0.573–0.988).

**Table 3:** Comparison of the ROC-AUC of the used ML strategies in different patient groups.

	All patients	AB naïvety	Patients with 2 SIRS criteria	Patients with 3 SIRS criteria	Patients with 4 SIRS criteria
*n*	466	380	182	213	71
Bacteraemia rate	28.8%	30.5%	28.6%	27.2%	33.8%
PCT	0.729 (0.679–0.779)	0.734 (0.680–0.787)	0.679 (0.598–0.762)	0.756 (0.678–0.833)	0.751 (0.633–0.869)
*rf*	0.738 (0.606–0.870)	0.727 (0.548–0.905)	0.698 (0.349–0.999)	0.781 (0.573–0.988)	0.585 (0.188–0.981)
*nn*	0.698 (0.549–0.857)	0.688 (0.499–0.876)	0.640 (0.355–0.925)	0.714 (0.497–0.930)	0.583 (0.181–0.985)
*en*	0.654 (0.493–0.815)	0.627 (0.396–0.858)	0.594 (0.334–0.854)	0.690 (0.466–0.914)	0.612 (0.214–0.999)

PCT = procalcitonin, *rf* = random forest, *nn* = neural network, *en* = elastic net.

## Discussion

Bacteraemia is a life-threatening condition, requiring prompt diagnostic and therapeutic actions. Due to the clinical similarities of symptoms of severe infections to inflammatory reactions not related to infections, treating physicians are faced with many uncertainties resulting in a low true positive result rate of BC analysis^20^.

In this study, we evaluated linear and non-linear algorithms for predicting bacteraemia in a relevant SIRS patient cohort with a high risk of bacteraemia (prevalence: 28.8%). Apart from PCT, several routinely and non-routinely available variables were evaluated, which presented a poor individual predictive capacity (see Table 2). Among the models tested, *rf* strategy led to the best performance, resulting in 0.738 ROC-AUC (95%CI: 0.606–0.870). Despite a moderate to low degree of correlation (see Fig. 1), inclusion of these variables did not improve the predictive capacity of PCT in *rf*-, *nn*- or *en*-based models.

In a systematic review published in 2015, fifteen publications on validated prediction systems on bacteraemia were found^22^. Amongst these, models for several infection-locus specific cohorts or hospital-specific cohorts were established and validated, including patients with community-acquired pneumonia (CAP^23–25^), patients with skin or skin structure infections^26^, female patients with pyelonephritis^27^, patients in the emergency department (ED^4,27–30^), hospitalized patients^19,31,32^ or ICU patients^29,33^. In 13 studies, logistic regression models were applied and in two studies Bayesian networks were implemented, resulting in ROC-AUCs between 0.60 and 0.83. Interestingly, none of these models were routinely applied at the time the review was published. Further, in only two studies was the predictive capacity of PCT for predicting bacteraemia evaluated. Müller *et al*. evaluated CAP patients and PCT resulted in 0.79 ROC-AUC using a validation cohort assessment (95%CI: 0.72–0.88)^23^. Unfortunately, only PCT was assessed and therefore the ability of other variables to increase the predictive capacity of PCT remained unevaluated. Tudela *et al*. used the Charlson co-morbidity index (≥2) and PCT (>0.4 ng/ml) to predict bacteraemia in patients in the ED^30^, yielding 0.80 ROC-AUC in the derivation cohort (*n* = 275) and 0.74 ROC-AUC in the validation cohort (*n* = 137).

Currently, the best validated prediction model was published by Shapiro for patients in ED^4^. In a prospective observational study with 3,901 patients (8.2% bacteraemia rate), a clinical prediction rule was established with 0.75 ROC-AUC in the validations set (*n* = 1,264). They stratified patients into three risk groups, the low-risk group showing a bacteraemia rate of 0.9% in the validation cohort. Thus, they concluded that for low-risk patients BC analysis might be omitted. In independent external validation studies, this rule resulted in similar ROC-AUCs^34,35^. Several similar scores and modifications of the Shapiro score have been established, resulting in a similar outcome^36–39^. Among these, in two independent studies a modified score including PCT was used, which performed better than PCT alone^38,39^. However, the generalizability of these results remains unclear, since in both studies a formal validation strategy was lacking.

Despite multiple pathophysiological differences on the cellular level, one might speculate that the host inflammation response to non-infectious stimuli is controlled similarly to the reaction to invasive pathogens. However, PCT presented with a higher diagnostic capacity in studies conducted at the ICU than on the standard care ward, as shown in a meta-analysis by Hoeber *et al*.^21^. They included data from our group as well^40^. On mixed standard care wards, the pooled sensitivity was 0.76 (95% CI: 0.65–0.85) and specificity was 0.66 (95% CI: 0.57–0.76) when using a 0.5 ng/ml cut-off value.

Since our patient cohort presented with a high degree of comorbidities, CRP or fibrinogen as acute phase reaction mediators were also high in non-bacteraemic SIRS patients. Thus, CRP was not useful as a bacteraemia marker. In a cohort of 785 CAP-patients with 4.5% bacteraemic patients, the PSI score (Pneumonia Severity Index for CAP, ROC-AUC: 0.720, 95%CI: 0.630–0.809) and the CURB-65 score (Confusion, BUN > 7 mmol/l, Respiratory rate ≥30, SBP <90 mmHg, DBP ≤ 60 mmHg, Age ≥ 65, ROC-AUC: 0.720; 95%CI: 0.622–0.819) showed a better capacity for predicting bacteraemia than CRP (ROC-AUC: 0.629, 95%CI: 0.522–0.735)^41^.

Further, a large proportion of SIRS patients presented with an infection, but without evidence of bacteraemia putatively contributing to the low predictive capacity of CRP. Interestingly, several liver-related blood markers presented a better predictive capacity than CRP for identification of bacteraemia. Our patient cohort was also stratified into risk groups according to the number of SIRS criteria fulfilled; however, the results were less convincing (see: Table 3). Generally, risk group stratification might have performed better when applying it in less specifically selected patients than our SIRS patients^4,32,42^. This might be based on the fact that SIRS criteria themselves are partly used for risk group stratification and therefore a further selection of low-risk patients was precluded. A similar observation was also found in CAP patients^43^.

In our study cohort, we found a relative heterogeneity in the patients’ co-morbidities, with a focus on oncological and haematological patients (see: Table 1), as described in^40,44,45^. Increased homogeneity might have led to better classification performance. Further, the study was performed in a single centre setting, and thus our negative finding is not necessarily generalizable to other settings. Because of this negative finding, an external validation strategy was not applied. Furthermore, since only a limited number of patients were available, we did not use any statistical variable selection strategies, which would have required an additional validation loop (e.g. nested CV)^46^. However, we applied methods that inherently face the inclusion of non-informative variables by penalization terms or weights. Moreover, within the imputation process, training data and test data sets were imputed at once with respect to their outcome, which could have led to over-optimistic results. However, this effect was considered to be limited, due to the relatively low number of total missing values.

PCT was the best individual marker for predicting bacteraemia in SIRS patients treated on standard care wards with having a moderate diagnostic accuracy. Combinations of clinical variables, various cytokines and routinely available laboratory markers using linear or non-linear machine learning algorithms failed to improve the diagnostic accuracy of PCT. Therefore, we concluded that machine learning models failed to improve the predictive capacity of PCT for identifying bacteraemia in our SIRS patient cohort.

## Methods

### Study design

The prospective cohort study was performed between July 2011 and September 2012 on 14 medical and 13 surgical standard care wards at the Vienna General Hospital, Austria. After approval by the ethics committee of the Medical University of Vienna (EC-No. 518/2011), the study was conducted in accordance with the Declaration of Helsinki 1964 (including current revisions) and the Good Clinical Practice guidelines of the European Commission. Prior to participation, all patients gave written informed consent. As describe elsewhere^40,44,45,47^, patients from whom a blood culture analysis was requested were screened for fulfilling at least two SIRS criteria, as defined by^48^. Neutropenia induced by chemotherapy was not considered an admissible SIRS criterion. Patients after surgical procedures were only included, when SIRS was developed 72 hours after surgery. Bacteraemia was specified by a positive BC or real-time multiplex polymerase chain reaction (PCR) analysis result for a recognized bacterial species. Bacterial contaminants were defined as described by Hall and Lyman^49^. Coagulase-negative staphylococci (CNS) were considered as causative pathogens only when detected in two blood specimens taken in separate venepunctures. Further, the infection status of all patients was assessed after discharge from hospital by applying the definition criteria for hospital-acquired infections, established by the European Centre of Disease Control (ECDC^50^,). A total of 3,370 patients with suspected bacteraemia were screened. In 2,750 patients, less than two SIRS criteria were observed and 154 patients met at least one exclusion criterion.

### Data collection

Clinical data was recorded during patients’ enrolment in this study, and was complemented after hospital discharge. Blood samples were cultured in a set of FA Plus (aerobic) and FN Plus (anaerobic) bottles using the BacT/ALERT 3D automated blood culture system (bioMérieux, Marcy l’Etoile, France). Bacterial isolates were specified by matrix-assisted laser desorption ionisation (MALDI) time of flight (TOF) mass spectroscopy (MS) using microflex LT with the Biotyper database (Bruker Daltonik GmbH, Bremen, Germany). In the event of Streptococcus pneumoniae identification, the assay result was additionally verified by optochin disc tests. Additionally, occurrence of microbial DNA was evaluated by the SeptiFast MGRADE test, which was applied in 220 patients according to the manufacturer’s specifications, as described in^47^.

The following 21 blood variables were analysed: procalcitonin (PCT, ng/ml, Hoffmann-La Roche Ltd, Basel, Switzerland), lipopolysaccharide-binding protein (LBP, µg/ml, IMMULITE 2000 Immunoassay System, Siemens Healthcare, Erlangen, Germany), C-reactive protein (CRP, mg/dl, Latex test; Beckman Coulter, Brea, CA, USA), interleukin-6 (IL-6, pg/ml, Hoffmann-La Roche Ltd), and fibrinogen according to Clauss (Fib, mg/dl, Hoffmann-La Roche Ltd, Basel, Switzerland). Further, albumin (Alb, g/l), alanine transaminase (ALAT, U/L), bilirubin (Bili, mg/dl), creatinine (Crea, mg/dl), gamma-glutamyl transpeptidase (γ-GT, U/L), serum iron (SI, µg/dl), lactate dehydrogenase (LDH, U/L), and total protein (TP, g/l; all reagents by Beckman Coulter, Brea, CA, USA) were analysed as standard laboratory parameters. Variables of the complete blood count including white blood cell counts (WBC, G/l), haemoglobin (Hb, g/dl); platelets (G/l), relative proportion of neutrophils (NeuR, %) and eosinophils (EosR, %) were analysed using a Stromatolyser-4DS (Sysmex, Norderstedt, Germany).

### Analysis of none-routinely available cytokines

In a screening phase, the following panel of 13 pro- and anti-inflammatory cytokines were analysed in 36 SIRS-patients (including 19 bacteraemic patients): epithelial-derived neutrophil-activating protein (ENA)−78, granulocyte-colony stimulating factor (G-CSF), interleukin (IL)1-Ra, IL1-b, IL-2, IL-4, IL-5, IL-8, IL-10, IL-17a, monocyte chemoattractant protein (MCP)-1, macrophage inflammatory protein (MIP)-1a, MIP-1b. In a second phase, the three markers with the highest predictive capacity (IL-10, (pg/mL), IL-17a (pg/mL), and MIP-1b (macrophage inflammatory protein-1β, pg/ml)) were quantified in all available patients. The human performance kit B (R&D Systems, Thermo Fisher Scientific, Waltham, USA) was used with the Luminex 200™ System (Luminex Corporation, Austin, USA) according to manufacturer’s specifications.

### Machine learning process

Machine learning methods were performed using R (version 3.3.0, Vienna, Austria^51^,). The caret package was used for model tuning and validation^52^. Random forest (*rf*, random forest package) and neural network models (*nn*) were used as non-linear models and compared to elastic net regression (*en*) as a linear model. Prior to model training, numerical data was standardized (Z-score standardization). The *rf* implementation described by Breimann was used with a maximum of 1,000 trees^53^. A single-hidden layer feedforward neural network, implemented in the nnet package, was used to establish the *nn* model^54^. During the model tuning process, the number of hidden units ranged from 1 to 10, the weight decay was set to 0, 0.1, 1 or 2, the maximum number of weights was set to 380 and the maximum number of iterations was set to 2,000. The following tuning parameters were used for the *en* model^55^: α from 0 to 1 (eight equidistant values, 0 = ridge regression, 1 = lasso regression), lambda from 0.1 to 1 (ten equidistant values).

Prior to the machine learning process, group differences between patients with or without bacteraemia were compared by using Fisher’s exact test or the Mann-Whitney U-test. Further, Spearman’s rank correlation coefficient (r_s_) was used to analyse the amount of correlation between variables. Statistical significance is defined as p-values less than 0.05 (two-tailed). An alpha accumulation error related to multiple testing was corrected by applying the Bonferroni-Holm correction.

The predictive capacity of individual variables was examined by comparing the area under the receiver operating curve (ROC-AUC). Missing data patterns were graphically assessed using the missing aggregation plot (VIM package). Multiple imputation (MI) was used for missing data imputation, using the mice package^56^. For imputation of numerical data, a predictive mean matching algorithm was applied, and ordinal or nominal data was imputed using logistic regression. Fifty completed data sets were generated.

Models were tuned using the training sets with a ten-fold cross validation (CV) scheme, repeated ten times. Among competing models, the model with the highest ROC-AUC was chosen. Prior to model training, study patients were randomly allocated to the training or test cohort using an 80:20 ratio (repeated ten times). For this split, bacteraemia status was used as a stratification criterion. Model prediction results of each patient were averaged over all imputed data sets in test set validation. This process was repeated ten times, resulting in different training sets and test sets for each repeat. The resulting ROC-AUCs were averaged over these ten repeats and the 95% confidence intervals (95% CI) of the ten repeats were calculated as follows: $\pm 1.96\sqrt{\bar{{variance}_{within}}+{variance}_{between}}$

### Availability of materials and data

Data cannot be made openly available to protect the privacy of participants. Further information about the data and conditions for access to anonymized data can be requested from the corresponding author.

## Electronic supplementary material

Supplementary table 1 and 2

## References

[1] Laupland KB (2013). Defining the epidemiology of bloodstream infections: the ‘gold standard’ of population-based assessment. Epidemiol Infect..

[2] Nielsen SL (2016). The daily risk of bacteremia during hospitalization and associated 30-day mortality evaluated in relation to the traditional classification of bacteremia. Am J Infect Control..

[3] Søgaard M, Nørgaard M, Dethlefsen C, Schønheyder HC (2011). Temporal changes in the incidence and 30-day mortality associated with bacteremia in hospitalized patients from 1992 through 2006: a population-based cohort study. Clin Infect Dis..

[4] Shapiro NI, Wolfe RE, Wright SB, Moore R, Bates DW (2008). Who needs a blood culture? A prospectively derived and validated prediction rule. J Emerg Med..

[5] Yahav D, Eliakim-Raz N, Leibovici L, Paul M (2016). Bloodstream infections in older patients. Virulence..

[6] Chase M (2012). Predictors of bacteremia in emergency department patients with suspected infection. Am J Emerg Med..

[7] Holmbom M (2016). 14-Year Survey in a Swedish County Reveals a Pronounced Increase in Bloodstream Infections (BSI). Comorbidity - An Independent Risk Factor for Both BSI and Mortality. PLoS one.

[8] Yang, C.-J. *et al*. The Impact of Inappropriate Antibiotics on Bacteremia Patients in a Community Hospital in Taiwan: An Emphasis on the Impact of Referral Information for Cases from a Hospital Affiliated Nursing Home. *BMC Infect Dis*. **13**, 10.1186/1471-2334-13-500 (2013). 10.1186/1471-2334-13-500 PMC4015527 24156241

[9] Kumar A (2006). Duration of hypotension before initiation of effective antimicrobial therapy is the critical determinant of survival in human septic shock. Crit Care Med..

[10] Westh H (2009). Multiplex real-time PCR and blood culture for identification of bloodstream pathogens in patients with suspected sepsis. Clin Microbiol Infect..

[11] Bloos F (2012). Evaluation of a polymerase chain reaction assay for pathogen detection in septic patients under routine condition: an observational study. PloS one.

[12] Perl B (1999). Cost-effectiveness of blood cultures for adult patients with cellulitis. Clin Infect Dis..

[13] Roth A (2010). Reducing Blood Culture Contamination by a Simple Informational Intervention. J Clin Microbiol..

[14] Bates DW, Cook EF, Goldman L, Lee TH (1990). Predicting bacteremia in hospitalized patients. A prospectively validated model. Ann Intern Med..

[15] Pien BC (2010). The Clinical and Prognostic Importance of Positive Blood Cultures in Adults. Am J Med..

[16] Little JR, Trovillion E, Fraser V (1997). High frequency of pseudobacteremia at a university hospital. Infect Control Hosp Epidemiol..

[17] Alahmadi YM (2011). Clinical and economic impact of contaminated blood cultures within the hospital setting. J Hosp Infect..

[18] Zwang O, Albert RK (2006). Analysis of strategies to improve cost effectiveness of blood cultures. J Hosp Med..

[19] Bates DW, Goldman L, Lee TH (1991). Contaminant blood cultures and resource utilization. The true consequences of false-positive results. JAMA..

[20] Long B, Koyfman A (2017). Clinical Mimics: An Emergency Medicine-Focused Review of Sepsis Mimics. J Emerg Med..

[21] Hoeboer SH, van der Geest PJ, Nieboer D, Groeneveld ABJ (2015). The diagnostic accuracy of procalcitonin for bacteraemia: a systematic review and meta-analysis. Clin Microbiol Infect..

[22] Eliakim-Raz, N., Bates, D. W. & Leibovici, L. Predicting bacteraemia in validated models—a systematic review. *Clin Microbiol Infect*. **2**1, 295–301, 10.1016/j.cmi.2015.01.023. 10.1016/j.cmi.2015.01.023 25677625

[23] Muller F (2010). Procalcitonin levels predict bacteremia in patients with community-acquired pneumonia: a prospective cohort trial. Chest.

[24] Lee J (2014). Bacteremia prediction model using a common clinical test in patients with community-acquired pneumonia. Am J Emerg Med..

[25] Metersky ML, Ma A, Bratzler DW, Houck PM (2004). Predicting bacteremia in patients with community-acquired pneumonia. Am J Respir Crit Care Med.

[26] Lipsky BA (2015). Predicting Bacteremia among Patients Hospitalized for Skin and Skin-Structure Infections: Derivation and Validation of a Risk Score. Infect Control Hosp Epidemiol..

[27] Kim KS (2011). A simple model to predict bacteremia in women with acute pyelonephritis. J Infect..

[28] Sasaki S (2017). Development and Validation of a Clinical Prediction Rule for Bacteremia among Maintenance Hemodialysis Patients in Outpatient Settings. PloS one.

[29] Bates DW (1997). Predicting bacteremia in patients with sepsis syndrome. J Infect Dis..

[30] Tudela P (2010). Prediction of bacteremia in patients with suspicion of infection in emergency room. Medicina Clinica.

[31] Paul M (2006). Prediction of Bacteremia Using TREAT, a Computerized Decision-Support System. Clin Infect Dis..

[32] A new statistical approach to predict bacteremia using electronic medical records. *Scand J Infect Dis*. **45**, 672–680, 10.3109/00365548.2013.799287 (2013). 10.3109/00365548.2013.799287 23808716

[33] Mozes B, Milatiner D, Block C, Blumstein Z, Halkin H (1993). Inconsistency of a model aimed at predicting bacteremia in hospitalized patients. J Clin Epidemiol..

[34] Jessen MK (2016). Prediction of bacteremia in the emergency department: an external validation of a clinical decision rule. Eur J Emerg Med..

[35] Hodgson LE, Dragolea N, Venn R, Dimitrov BD, Forni LG (2016). An external validation study of a clinical prediction rule for medical patients with suspected bacteraemia. Emerg. Med. J..

[36] Takeshima T (2016). Identifying Patients with Bacteremia in Community-Hospital Emergency Rooms: A Retrospective Cohort Study. PloS one.

[37] Brown JD, Chapman S, Ferguson PE (2017). Blood cultures and bacteraemia in an Australian emergency department: Evaluating a predictive rule to guide collection and their clinical impact. Emerg. Med. Australas..

[38] Lee C-C (2012). Prediction of community-onset bacteremia among febrile adults visiting an emergency department: rigor matters. Diagn Microbiol Infect Dis..

[39] Laukemann S (2015). Can We Reduce Negative Blood Cultures With Clinical Scores and Blood Markers? Results From an Observational Cohort Study. Medicine.

[40] Ratzinger F (2013). Utility of sepsis biomarkers and the infection probability score to discriminate sepsis and systemic inflammatory response syndrome in standard care patients. PloS one.

[41] Lee JH, Kim YH (2016). Predictive factors of true bacteremia and the clinical utility of blood cultures as a prognostic tool in patients with community-onset pneumonia. Medicine.

[42] Ratzinger F (2014). A Risk Prediction Model for Screening Bacteremic Patients: A Cross Sectional Study. PloS one.

[43] van Werkhoven CH, Huijts SM, Postma DF, Oosterheert JJ, Bonten MJM (2015). Predictors of Bacteraemia in Patients with Suspected Community-Acquired Pneumonia. PloS one.

[44] Ratzinger F (2015). Sepsis in standard care: patients’ characteristics, effectiveness of antimicrobial therapy and patient outcome–a cohort study. Infection.

[45] Krstajic D, Buturovic LJ, Leahy DE, Thomas S (2014). Cross-validation pitfalls when selecting and assessing regression and classification models. J Cheminform..

[46] Ratzinger F (2015). Sepsis biomarkers in neutropaenic systemic inflammatory response syndrome patients on standard care wards. Eur J Clin Invest..

[47] Ratzinger F (2016). Evaluation of the Septifast MGrade Test on Standard Care Wards-A Cohort Study. PloS one.

[48] Bone, R. C. *et al*. Definitions for sepsis and organ failure and guidelines for the use of innovative therapies in sepsis. The ACCP/SCCM Consensus Conference Committee. American College of Chest Physicians/Society of Critical Care Medicine. *Chest* **101**, 1644-1655 (1992). 10.1378/chest.101.6.1644 1303622

[49] Hall KK, Lyman JA (2006). Updated review of blood culture contamination. Clin Microbiol Rev..

[50] European Centre for Disease Prevention and Control, 2012. Point prevalence survey of healthcare- associated infections and antimicrobial use in European acute care hospitals – protocol version 4.3. ECDC, Stockholm, ISBN: 9789291933662, 10.2900/5348

[51] R Development Core Team 2008. R: A language and environment for statistical computing. R Foundation for Statistical Computing, Vienna, Austria. ISBN 3-900051-07-0, http://www.R-project.org [22.02.2018]

[52] Kuhn, M. Building Predictive Models in R Using the caret Package. *J Stat Soft* 2**8**, *Issue 5* (2008).

[53] Breiman L (2001). Random Forests. Machine Learning.

[54] Venables, W. N. & Ripley, B. D. *Modern Applied Statistics with S*. Springer-Verlag New York. ISBN: 978-0-387-95457-8, pp 2011–250 (2010).

[55] Friedman J, Hastie T, Tibshirani R (2010). Regularization Paths for Generalized Linear Models via Coordinate Descent. Journal of statistical software.

[56] van Buuren, S. & Groothuis-Oudshoorn, K. mice: Multivariate Imputation by Chained Equations in R. *J Stat Soft* **45** (2011).

