# Supplementary material for: Machine learning for fast identification of bacteraemia in SIRS patients treated on standard care wards: a cohort study
Source: Sci Rep. 2018 Aug 15;8:12233. doi: 10.1038/s41598-018-30236-9 (PMC6093921; doi:10.1038/s41598-018-30236-9)
Supplement: Supplementary file 1 — Supplementary table 1 and 2 [file 41598_2018_30236_MOESM1_ESM.pdf]

Supplementary information: Machine learning for fast identification of bacteraemia in SIRS patients treated on standard care wards: a cohort study

Franz Ratzinger[1], Helmuth Haslacher[1], Thomas Perkmann[1], Matilde Pinzan[1], Philip Anner[2], Athanasios Makristathis[3], Heinz Burgmann[4], Georg Heinze[5], Georg Dorffner[2]*

[1] Department of Laboratory Medicine, Division of Medical and Chemical Laboratory Diagnostics, Medical University of Vienna, Vienna, Austria
[2] Center for Medical Statistics, Informatics and Intelligent Systems, Section for Artificial Intelligence and Decision Support, Medical University of Vienna
[3] Department of Laboratory Medicine, Division of Clinical Microbiology, Medical University of Vienna, Vienna, Austria
[4] Department of Medicine I, Division of Infectious Diseases and Tropical Medicine, Medical University of Vienna, Vienna, Austria
[5] Center for Medical Statistics, Informatics, and Intelligent Systems; Section for Clinical Biometrics, Medical University of Vienna, Vienna, Austria

Supplementary table 1: Distribution of infections according to ECDC classification criteria modified ECDC class according to [50]

Type	ECDC class	n	%
**Bloodstream infection[1]**	C-CVC[3] (n=20), S-DIG[4] (n=20), S-PUL[5] (n=14), S-SSI[6] (n=9), S-SST[7] (n=4) S-UTI[8] (n=12), S-OTH[9] (n=21), S-UO[10] (n=34)	134	
**Respiratory tract infection[2]**	PN1[11] (n=3), PN3[12] (n=6), PN4[13] (n=5), PN5[14] (n=61), LRI-Bron[15](n=3), LRI-Lung[16](n=2)	80	
**Gastrointestinal system infection[2]**	GI-CDI[17] (n=5), GI-GE[18] (n=5), GI-GIT[19] (n=7), GI-IAB[20] (n=11), EENT-ORAL[21] (n=2)	30	
**Urinary tract infection[2]**	UTI-A[22] (n=18), UTI-B[23] (n=21)	39	
**Others[2]**	SYS-CESP[24] (n=7), SYS-DI[25] (n=7), SSI-S[26] (n=9), SSI-D[27] (n=1), SSI-O[28] (n=2), CVS-Card[29] (n=2), CVS-Vasc[30] (n=1), CVS-Endo[31] (n=2), SST-Skin[32] (n=4), SST-ST[33] (n=3), REPR-OREP[34] (n=2), CRI-CVC[35](n=3), CNS-IC[36] (n=1), CNS-SA[37](n=1), BJ-JNT[38] (n=1)	46	
**Total**		329	100%

Type = type of infection, [1]= blood culture positive; [2]= blood culture negative; [3]= blood stream infection (BSI), related to central vascular catheter; [4]= BSI, secondary digestive tract infection; [5]= BSI, secondary to pulmonary infection; [6]= BSI, secondary to surgical site infection; [7]= BSI, secondary to skin and soft tissue infection; [8]= BSI,

secondary to urinary tract infection; [9]= BSI, secondary to another infection; [10]= BSI, (confirmed) unknown origin; [11]= pneumonia, positive quantitative culture from minimally contaminated lower respiratory tract specimen; [12]= pneumonia, microbiological diagnosis by alternative microbiology methods, [13]= pneumonia, positive sputum culture or non-quantitative culture from lower respiratory tract specimen; [14]= pneumonia, clinical signs of pneumonia without positive microbiology; bronchitis, tracheobronchitis, bronchiolitis, tracheitis, without evidence of pneumonia; [15]= LRI, other infections of the lower respiratory tract, bronchitis, tracheobronchitis, bronchiolitis, tracheitis; [16]= lower respiratory tract infection, other than pneumonia; [17]= gastrointestinal system infections (GI) *clostridium difficile* infection; [18]= GI, gastroenteritis (excluding CDI); [19]= Gastrointestinal tract (oesophagus, stomach, small and large bowel, and rectum), excluding GE, CDI; [20]= GI, intra-abdominal infection, not specified elsewhere; [21]= eye, ear, nose or mouth infection (EENT), oral cavity (mouth, tongue, or gums); [22]= urinary tract infection (UTI), microbiologically confirmed symptomatic UTI; [23]= UTI, not microbiologically confirmed symptomatic UTI; [24]= systemic infections (SYS), clinical sepsis in adults and children; [25]= SYS, disseminated infection; [26]= surgical site infection (SSI), superficial incisional; [27]= surgical site infection (SSI), deep incisional;[28]= surgical site infection, organ/space; [29]= cardiovascular system infection (CVS), myocarditis or pericarditis; [30] = CVS, arterial or venous infection; [31]= endocarditis, [32]= skin and soft tissue infections (SST), skin; [33]= SST, soft tissue (necrotizing fasciitis, infectious gangrene, necrotizing cellulitis, infectious myositis, lymphadenitis, or lymphangitis); [34]= reproductive tract infections (REPR)-other infections of the male or female reproductive tract (OREP), [35]= central vascular catheter-related infection (CRI), general CVC-related infection (no positive blood culture); [36]= central nervous system infection (CNS), intracranial infection; [37]= CNS-SA: spinal abscess without meningitis; [38]BJ-JNT= joint or bursa infection

Supplementary table 2: Distribution of pathogens detected by BC and Septifast analysis

	Pathogen	BC*	Septifast
Gram positive	*Staphylococcus aureus*	23	10
	Coagulasis negative staphylococcus[1]	13[A]	7
	Streptococcus pneumoniae	3	2
	Stretococcus pyogenes	3	3
	Streptococcus species[1]	6[B]	3
	Enterococcus faecalis	8	3
	Enterococcus faecium	7	3
	Lactobacillus fermentum	1	-
	Clostridium perfringens	-	1
	Corynebacterium urealyticum	1	-
Gram negative	*Escherichia coli*	30	5
	Pseudomonas aeruginosa	10	4
	Klebsiella pneumoniae[2]	10	7
	Klebsiella oxytoca[2]	2	
	Citrobacter species	4[C]	-
	Serratia marcescens	2	-
	Enterobacter cloace complex[2]	1	3
	Proteus mirabilis	2	-
	Campylobacter jejuni	1	-
	Raoultella ornithinolytica	1	-
	Acinetobacter baumanii calcoaceticus	1	-
Fungi	*Candida albicans*	3	1
Total number of pathogens		132	52

*in 348 patients two or more sets of blood culture bottles were sampled; [1]Coagulase-negative staphylococci and streptococci (except S. pneumoniae) are identifiable at the genus level only by using the Septifast test; [2]Septifast cannot differentiate between *Klebsiella pneumoniae* and *Klebsiella oxytoca* as well as *Enterobacter cloacae* and *Enterobacter aerogenes*; [A]*Staphylococcus epidermidis*= 12, *Staphylococcus haemolyticus*= 1; [B] *Streptococcus mitis/oralis*= 2, *Streptococcus mutans*=1, *Streptococcus pluranimalium*=1,*Streptococcus agalactiae*= 1, *Streptococcus constellatus*=1; [C]*Citrobacter braakii*=1, *Citrobacter freundii*= 1, *Citrobacter koseri*= 2
